# In-Situ Incorporation of Alkyl-Grafted Silica into Waterborne Polyurethane with High Solid Content for Enhanced Physical Properties of Coatings

**DOI:** 10.3390/polym10050514

**Published:** 2018-05-09

**Authors:** Yanting Han, Jinlian Hu, Zhongyin Xin

**Affiliations:** 1 Institute of Textiles & Clothing, The Hong Kong Polytechnic University, Hung Hom, Kowloon 999077, Hong Kong, China; 16901226r@connect.polyu.hk; 2 National Engineering Laboratory for Clean Technology of Leather Manufacture, Sichuan University, Chengdu 610044, China; xinzyin@163.com

**Keywords:** waterborne polyurethane, silica, hybrid, high solid content, coatings, microphase

## Abstract

Waterborne polyurethane (WPU) with high solid content (45%) was obtained by utilizing dimethylol propionic acid (DMPA) and ethoxylated capped polymeric diol as complex hydrophilic groups. Alkyl-grafted silica was incorporated into polymer matrix through in situ polymerization to improve the performance of coatings casted from WPU dispersions. The addition of alkyl-grafted silica enlarged the particle size distribution whilst increased emulsion viscosity, which showed little influence on attainment of high solid content for WPU. The properties of obtained WPU/Silica coatings were investigated. Results showed that the functionalized surface of silica provides good compatibility with the WPU matrix, which promoted the homogeneous dispersion of silica particles. This facilitated the formation of nanosized silica papillae on coatings, contributing to surface roughness and hydrophobicity. Solvent resistance of WPU was enhanced with existence of alkyl-grafted silica particles. The WPU/Silica coatings also displayed improved thermal stability due to the thermal insulation ability and tortuous path effect of silica. Besides this, valid interactions between silica and WPU resulted in hybrid microphase of which the synergistic effect imparted superior mechanical properties at relatively low loadings of silica (2%). The facile technique presented here will provide an effective and promising method for preparing WPU hybrids with enhanced performance.

## 1. Introduction

Compared with traditional solvent-based polyurethane, waterborne polyurethane (WPU) has the advantages of being non-toxic, non-flammable, and non-polluting to the environment [1]. They have been widely used in leather finishing, automotive painting, fiber processing, coatings, and adhesives [2]. In WPU dispersions, polyurethane exists in water in the form of latex particles. The viscosity of WPU dispersions increases with the increase of solid content since, when the particles are crowded, the interactions between neighboring particles become strong. As high viscosity will lead to more burden of production and product handling equipment, conventional WPU usually has a low solid content in the range of 20% to 40%. The low solid content results in inferior drying rates and slower development of adhesion. These shortcomings can be largely overcome by increasing the solid content of WPU [3]. Hence, high solid content (40~55%) of WPU has received much attention from academic and industry fields [4,5,6]. For example, Kim et al. [7] have found that high solid content (45%) of WPU can be obtained at a low ionic content (2%) by incorporating the ionic groups to the soft segments. Shan et al. [4] used nonionic monomers (polyethylene glycol, PEG) together with anionic monomers (dimethylolpropionic acid, DMPA) as hydrophilic groups to synthesize WPU. The introduction of nonionic hydrophilic chains helps to decrease required dosage of DMPA, and thus to reduce the interaction force and hydration layer. Similarly, Wei et al. [6] proved that the complex hydrophilic chain-extending agent led to the multimodal distribution of latex particles. As a result, large particles and small particles can compact tightly, increasing the maximum packing factor of particles.

Although most of the literature claim that high solid content of WPU can be achieved by molecular design, their results revealed that the stability of the WPU may be reduced; especially, the performance of casting films could be impaired [6]. However, it is addressed that the excellent properties of organic/inorganic hybrids can provide efficient methods and approaches for high performance and functionalization of various materials based on the synergistic effect [8]. Particularly, as one of the important reinforcing fillers, hybrid with silica has been reported as an alternative approach to improving the properties of final products. For example, Malaki et al. [9] added silica into acrylic polyurethane to fabricate hybrid coatings. Their results showed that the addition of silica is beneficial for the adhesive strength and erosion resistance. Li et al. [10] concluded that the silica additives significantly improved the hardness and abrasion with uniform dispersion of silica nanoparticles. Wang et al. [11] prepared the polyurethane/silica hybrids via chemical reaction between urethane groups of PU prepolymer and hydroxyl groups at the surfaces of silica. They found that silica can work as inorganic hard-segment which contributes to the enhanced mechanical properties of hybrids. As all these literatures confirmed the reinforcement effect of silica, we can propose that adding silica to WPU should be an effective way to improve the performance of high solid content WPU. In this case, the challenge is to adopt appropriate methods to incorporating silica into WPU dispersions without affecting the achievement of high solid content. Generally, there are three ways for adding silica into PU matrix: blending method, sol-gel method, and in situ polymerization.

The blending method is the simplest method for preparing nanocomposites. However, silica particles are easily aggregate due to the large number of hydroxyl groups, making it difficult to evenly disperse silica in PU matrix [12]. To solve this problem, sol-gel methods consisting of hydrolysis and polycondensation were developed. Generally, the precursor is first dissolved in the monomer or prepolymer solution, then gradually hydrolyzed to generate nanosized silica particles with the aid of a catalyst [13]. This process can lead to an organic-inorganic network with uniformly dispersed silica particles. Nevertheless, the inevitable use of toxic precursors and solvents increases the production cost and causes environmental pollution. Most notably, the requirement of large amounts of solvent during sol-gel process violates the aim of high solid content for WPU. In contrast, in situ polymerization does not have strict requirements for the solvent. Since silica particles are first uniformly dispersed with prepolymers, the subsequent polymerization can occur around the nanoparticles, which facilitates the interactions between silica and polymer molecule [14,15]. This in turn can prevent the aggregation of silica particles. Moreover, research has proved that grafting alkyl monomers to the surface of silica can promote its miscibility with polymer components because of its decreased hydrophilicity and enhanced interfacial interaction between silica and PU matrix, through entanglement of the grafting alkyls attached to the silica particles with the polymer molecules [16,17].

Therefore, this study aims to improve the properties of coatings casted from WPU with high solid content through addition of alkyl-grafted silica as nanofillers. Here, WPU with high solid content (45%) was obtained by utilizing dimethylol propionic acid (DMPA) and ethoxylated capped polymeric diol as complex hydrophilic groups. To improve the performance of final coatings, alkyl-grafted silica was incorporated into the WPU matrix through in situ polymerization. The effect of alkyl-grafted silica on the resultant WPU/Silica dispersions as well as the morphology, solvent resistance, thermal stabilities, and mechanical properties of the hybrid coatings were investigated. This research should contribute to the synthesis of environment-friendly coatings with high performance.

## 2. Experiments

### 2.1. Materials

Isophorone diisocyanate (IPDI), purchased from Bayer (Leverkusen, Germany). Polytetrahydrofurfuryl glycol (PTMG-1K: 1000 g/mol; PTMG-2K: 2000 g/mol), dimethylol propionic acid (DMPA), pentaerythritol (PE), and ethoxylated capped polymeric diol (YmerTM N120) were obtained by Perstorp Specialty Chemicals (Perstorp, Sweden). Alkyl-grafted silica (Aerosil^®^ R816) was offered by Degussa Chemical (Essen‎, Germany). Dibutyltin dilaurate (T-12), Ethylenediamine (EDA), *N*,*N*-dimethylformamide (DMF) were produced from Chengdu Kelong Chemical Reagent Factory (Chengdu, China). DMPA and PE were dried in vacuum at 100 °C for 6 h before use. Laboratory-made distilled water and other reagents were used without treatment.

### 2.2. Preparation of Waterborne Polyurethane/Silica (WPU/Silica) Hybrid Dispersions with High Solid Content

**Figure 1:**
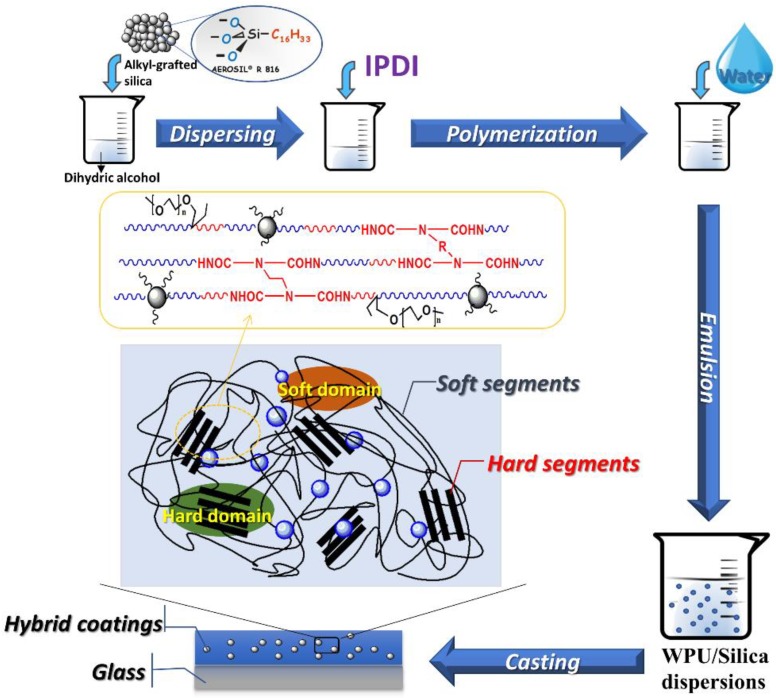
Preparation of waterborne polyurethane/Silica (WPU/Silica) dispersions with high solid content and their coatings.

PTMG-1K (0.0450 mol), PTMG-2K (0.0225 mol), DMPA (0.0138 mol), ethoxylated capped polymeric diol (0.0120 mol), and quantitative long-chain alkyl modified silica were added to a three-necked flask equipped with a stirrer and a temperature controller. The mixture was dispersed at a high speed stirring with temperature raising to 110 °C. The dehydration was performed under a negative pressure of 0.08 MPa for 90 min. After cooling to 40 °C, IPDI (0.200 mol) was added followed by the addition of catalyst (0.5 wt %). Then, the temperature was raised to 90 °C for full reaction. During the reaction, the free –NCO content was monitored by the standard dibutylamine back-titration method. Upon reaching the desired –NCO value, DMPA was added and the reaction was continued at 90 °C until the NCO content reached the theoretical value. Subsequently, the temperature was lowered to 70 °C and 1 wt % PE (0.0111 mol) was added, which is followed by slowly raising the temperature to 90 °C. When the –NCO content reached the theoretical value, the pre-polymer was cooled down to ~60 °C, followed by the addition of triethylamine (0.0130 mol). After neutralization for 10 mins, the temperature of pre-polymer was decreased to ~40 °C. Then, quantitative deionized cold water (5 °C) was rapidly added for emulsification. This process was done in an ice bath for 10 min under vigorous stirring. The pH value of dispersion was ~7. Diluted EDA (0.0598 mol) was then slowly added dropwise for chain extension for 1.5 h at 35 °C. The high solid content (~45%) was measured according to the method described by Gong et al. [3]. Coatings from WPU/Silica were prepared by casting dispersions onto the glass plate at ambient temperature for 5 days and 60 °C for 24 h The obtained coatings with thickness of ~0.4 mm was stored in a desiccator (Hangzhou Kebo Instrument CO., LTD, Hangzhou, China) for later testing. Figure 1 outlines the in situ polymerization of waterborne polyurethane/Silica (WPU/Silica) dispersions and preparation of coatings. According to the mass fraction of the silica, the samples were designated as WPU/Silica-0.5, WPU/Silica-1.0, WPU/Silica-1.5, WPU/Silica-1.8, WPU/Silica-2.0, and WPU/Silica-2.3. For example, WPU/Silica-1.0 indicates that the addition of silica accounts for 1.0% of the total mass of the resin. Meanwhile, as comparison, pure WPU was blended with 1.0% silica to prepare a composite which was named as WPU/Silica-1.0-B.

### 2.3. Characterization

The mean particle size, particle size distribution, and zeta potential of dispersions were investigated by Zetasizer Nano S90 laser particle analyzer (Malvern Instrument, Malvern, UK) in polystyrene statistical model. Samples were diluted with deionized water before testing. The viscosities of dispersions were measured on NDJ-8S digital readout viscometer at 25 °C. The rotate speeds used were 3, 6, 12, 30, and 60 rpm. Fourier transform infrared (FTIR) spectra of the film samples were performed on a NEXUS 670 spectrophotometer (Nicolet, Madison, WI, USA) in transmission mode with a resolution of 4 cm^−1^ and 32 scanning times. The cross-section morphology of specimens was observed under a Phenom (Kunming, China) scanning electron microscopy (SEM) with an accelerating voltage of 5 kV. Atom force microscopy (AFM) was performed on the surface of the film prepared by casting 0.02 mL dispersions on mica plate. The measurement was conducted using the SPA-400 Atomic force microscope (Seiko Instruments Inc., Chiba, Japan) in tapping mode. Surface roughness analysis and particle size distribution were carried out using NanoScope-Analysis software (Veeco Instruments, Plainview, NY, USA). Contact angles of films with deionized water and glycol were determined using the OCAH200 high-speed video contact angle measurer (DataPhysics Instruments GmbH Ger, Filderstadt, Germany). Water and acetone swelling ratio of prepared coatings were examined by following equation:

Swelling (%) = (*W* − *W*_0_)/*W*_0_ × 100% (1)

where *W*_0_ is the original weight of dried sample (20 mm × 20 mm in size) and *W* is the weight of swollen sample after immersion in solvent at 25 °C for 24 h.

Differential Scanning Calorimetric (DSC) was performed on DSC200 (NETZSCH Instruments, Ahlden, Germany) at a heating rate of 5 °C/min from −100 to 150 °C under nitrogen atmosphere. Thermogravimetric analysis (TGA) was carried out by a differential thermogravimetric analyzer (NETZSCH Instruments, Germany) at a heating rate of 10 °C/min from 100 to 550 °C under nitrogen atmosphere. Dynamic viscoelastic properties of samples were investigated using DMA 242 C analyzer (NETZSCH Instruments, Germany) with a heating rate 5 °C/min from −100~150 °C at 1 Hz in stretching mode with a amplitude of 30 μm. Mechanical properties of samples with a rectangle size of 5 mm × 25 mm were measured using GT-AI-7000S tensile testing machine (Gotech Testing Machines Inc., Guangzhou, China) with a stretching speed of 100 mm/min under room temperature.

## 3. Results and Discussion

### 3.1. Characterization of the Waterborne Polyurethane/Silica Dispersions (WPU/Silica) with High Solid Content

**Figure 2:**
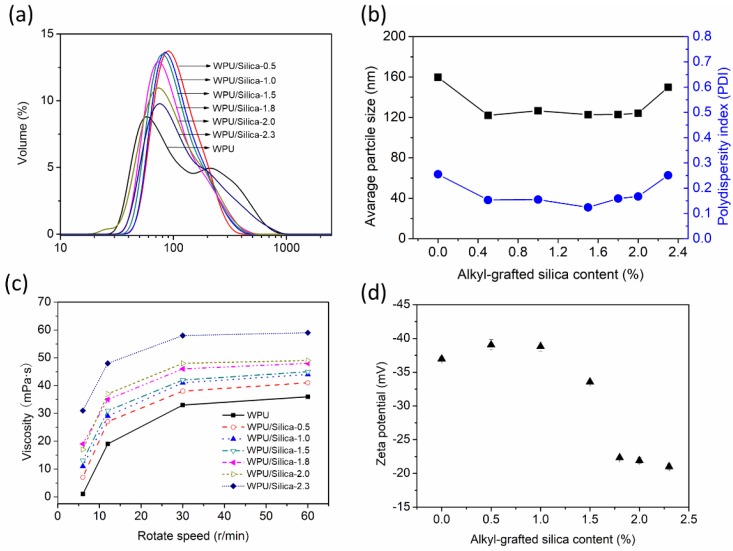
Emulsion properties of high solid content of WPU and WPU/Silica dispersions: (**a**) particle size distribution; (**b**) average particle size and polydispersity index (PDI); (**c**) Zeta values; (**d**) rheological properties.

The particle size distributions of the WPU/Silica dispersions are shown in Figure 2a. The mean particle size of the WPU/Silica decreased at low content of Silica (<2.0%) compared to WPU, at the same time the particle size distribution of WPU/Silica became narrow. It can be seen from Figure 2b that the average particle size of WPU/Silica was around 122 nm, while the polydispersity index (PDI) decreased first and then increased with augment of silica content (>2.0%). The initial decline of PDI may be because the strong rigidity of silica nanoparticle can accelerate the dispersion efficiency through the physical friction effect when the prepolymer matrix is emulsified [18]. It is suggested that the hydrophobic parts of WPU chains will be surrounded by hydrophilic segments due to energetic reasons in WPU dispersions [4,6]. Hence, the alkyl-grafted silicas are supposed to be shielded by more hydrophilic chains of WPU. Thus, with incorporation of the higher content silica, the particle size of WPU/Silica enlarges, which may be due to the increased number of silica packaged by WPU chains. The wide particle size distribution is also beneficial for high solid content, because small particles can pack into the voids among the large particles. In this case, the maximum packing factor of particles in water can be improved [5].

Viscosity of prepolymer is also reported to influence the particle size [19]. During our preparation of WPU/Silica, the prepolymer viscosity increased as the content of alkyl-grafted silica increased. Byung et al. [20] found that the polymer viscosity directly contributed to the finer breakup of the dispersed phase during phase version with water addition, in agreement with our results. The viscosities of WPU/Silica dispersions at different rotate speeds are shown in Figure 2c. Results showed that WPU/Silica dispersions were non-Newtonian fluids with viscosity increased with rotate speed, and the viscosity of WPU/Silica increased as the content of silica increases. With larger particle size containing higher content of silica, dispersions should give greater viscosity, owing to the greater volume fraction of fillers according to [21]:

ln (η/η_1_) = (*K*_E_/*ϕ*)/(1 − *ϕ*/*ϕ*_m_) (2)

where η is the viscosity of component containing fillers, η_1_ is s the viscosity of neat component. *K*_E_ is the Einstein constant (2.5), *ϕ* is the volume fraction of component containing fillers, and *ϕ*_m_ is maximum value of *ϕ*. *ϕ* increases as the mass fraction of filler increases. This leads to the increased viscosity of the WPU containing silica particles. On the other hand, the large specific surface area of silica nanoparticles can promote the adsorption of water by silica to increase the volume of the dispersed phase, as a result, the viscosity of the emulsion increases.

Zeta potential is one of the key parameters for evaluation of the stability of emulsions [7]. The higher value of zeta potential confers the stability due to the strong repellant between emulsion particles. It can be seen from Figure 2d that the absolute value of the zeta potential of WPU/Silica showed an increase with increasing the silica content to 1.0%, indicating the enhanced stability of WPU/Silica dispersion. Nevertheless, the decrease of zeta potential is probably because the incorporation of silica increases the particle size of the emulsion, hence, the density of carboxyl groups per unit volume of emulsion particle decreases. In other words, the number of charge per unit volume decreases, thereby causing the decline of zeta potential.

### 3.2. Structure and Morphology of WPU/Silica Coatings

**Figure 3:**
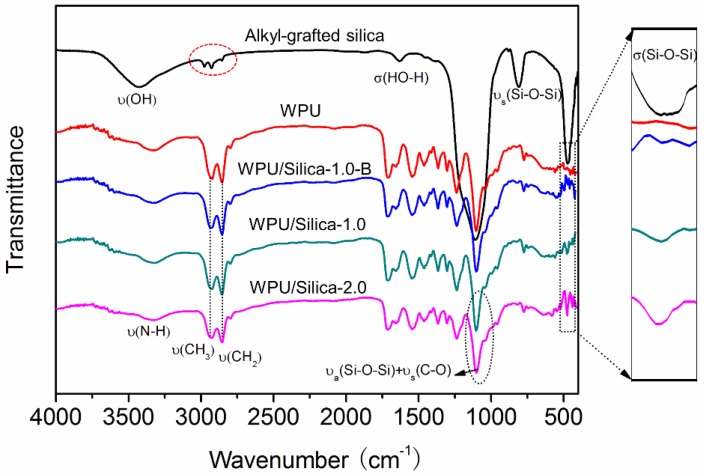
FTIR spectra of alkyl-grafted silica, WPU, and WPU/Silica.

FTIR spectra of alkyl-grafted silica, pure WPU, and WPU/Silica coatings are presented in Figure 3. In the infrared spectrum of alkyl-grafted silica, stretching absorption peaks of Si–O–Si were observed at 1100 and 808 cm^−1^. The absorption peaks in the range of 3200~3750 cm^−1^ belongs to the vibration absorption of Si–OH and absorbed water while the peak at 940 and 2850 cm^−1^ attribute to stretching vibrations of –CH_2_ and –CH_3_ respectively [22]. These peaks confirmed the silica structure with alkyl groups on surface. Notably, there was a peak at 480 cm^−1^ on spectrum of WPU/Silica specimens due to the vibration of Si–O–Si. This peak was also found on the spectrum of WPU/Silica specimens while WPU had no characteristic peak of Si–O–Si. It can be seen from the enlarged part that the intensity of the peak enhanced with increasing silica content. Compared to WPU/Silica-1.0, WPU/Silica-1.0-B had a weak peak for Si–O–Si at 480 cm^−1^. This may be because the physical blending method causes the unstable silica particles in WPU dispersions. Hence, silica particles may agglomerate in a block rather than be evenly distributed during the formation process of coatings. Moreover, the peak of Si–OH disappeared in spectrum of WPU/silica specimens. This may be due to the bonding effect of Si–OH to the polyurethane segments [23,24]. At the same time, the intensity of peak at 1100 cm^−1^ showed an increase with incorporation of silica due to the overlapping absorption peak of C–O–C with Si–O–Si [9,11]. Hence, it can be concluded that alkyl-grafted silica was successfully incorporated into WPU matrix by in situ polymerization.

**Figure 4:**
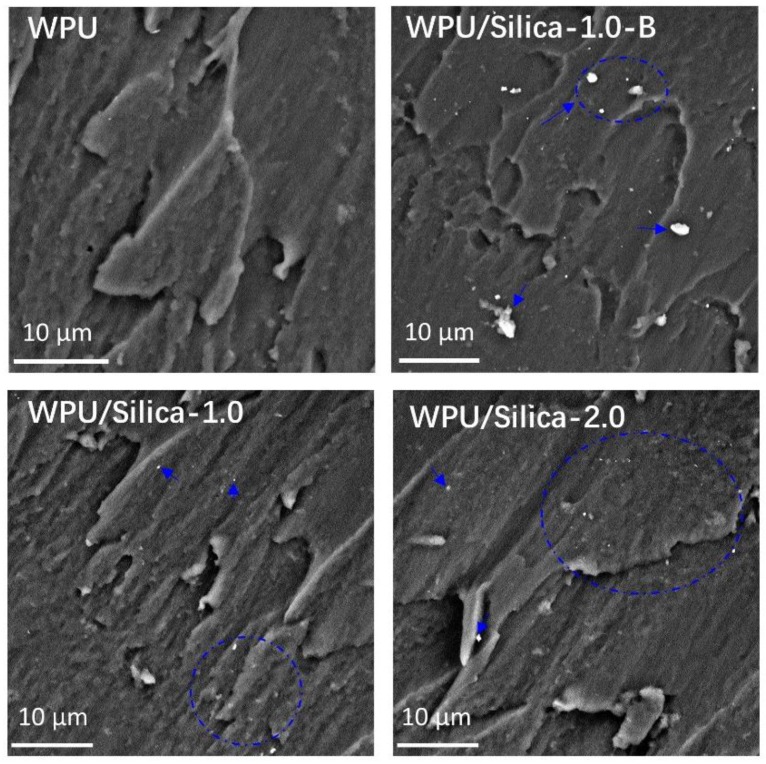
SEM images of cross sections of WPU and WPU/Silica coatings.

If the fillers are not uniformly dispersed in the emulsion, agglomeration will occur inside the formed coating. Hence, the presence and distribution of fillers in emulsions matrix can be evaluated by their dispersion in coating through SEM study [25]. Figure 4 shows the SEM micrographs of the cross sections of the prepared samples. Compared to neat WPU, alkyl-grafted silica particles were observed in WPU/Silica coatings regardless of the preparation method. However, the blending method basically caused obvious aggregation at 1% loading of silica (see samples WPU/Silica-1.0-B). On the contrary, the in situ method resulted in an even dispersion of silica for WPU/Silica-1.0 and WPU/Silica-2.0, which indicates that in situ incorporation is conducive to uniform dispersion of silica particles. Such an even and uniform distribution of the fillers in the matrix was reported to play an important role in improving the mechanical performance of the composite coatings [9].

**Figure 5:**
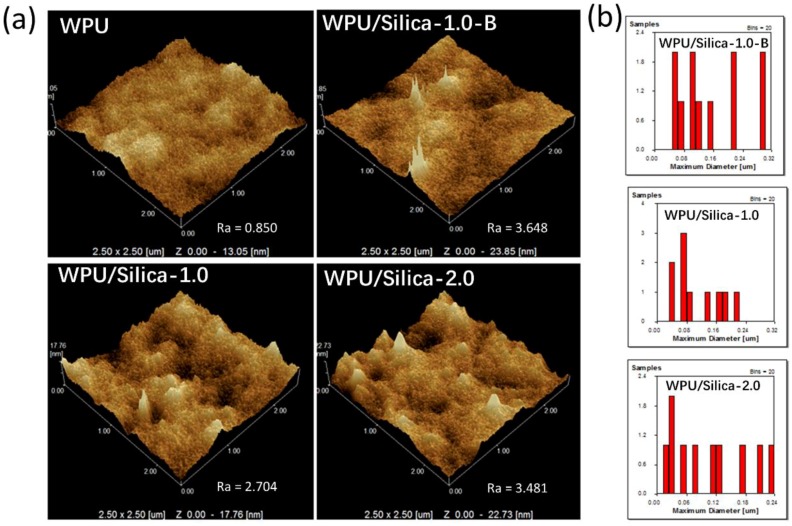
(**a**) 3D topography of WPU and WPU/Silica coatings; (**b**) particle distribution of WPU/silica.

AFM phase images (Figure S1) illustrate the typical microphase separation structure of WPU/Silica coatings. The 3D topography can demonstrate the surface properties of coatings. As shown in Figure 5, there were dots protruding out of the surface for WPU/Silica coatings. These papillae attribute to the alkyl-grafted silica particles embedded in WPU matrix, which indicates that silica particles can migrate to the film surface rather than totally deposit inside the WPU matrix during the film formation process. Hence, the surface properties of WPU/Silica coatings should be determined by the synergy of WPU and silica particles. With increasing silica content, the number of silica dots increased, notably, the average particle size for WPU/Silica ranged from 100~200 nm (Figure 5b) as analyzed by NanoScope-Analysis software (Veeco Instruments, USA). In comparison, silica particles in WPU/Silica-1.0-B had diameters larger than 300 nm. This is evidence that in situ polymerization can promote the interactions between the alkyl chains of silica and prepolymers, which stabilizes the silica particles in WPU dispersions, thereby preventing silica from cohesion and leading to better dispersion and uniformity. The results were consistent with the SEM analysis. In addition, silica content also influences the particle distribution. In the recent research of Xue et al. [26] about silica hybrid coatings, although distributions of particles were homogeneous with a load of 3% silica, distinct agglomeration were observed in the hybrids. This defection would impair the mechanical properties of coatings, which, however, was not discussed in their research. Taking a closer look at Figure 5b, there are no cracks and obvious boundaries between alkyl-grafted silica and WPU matrix due to their good compatibility, which benefits from the existence of polymer chains on the surface of silica. Surface roughness (*R*_a_) of coatings were also analyzed by AFM. Expectedly, surface roughness of coatings increased after addition of silica as *R*_a_ increased from 0.850 of WPU to 3.481 of WPU/Silica-2.0. Even so, the addition of alkyl-grafted silica showed little influence on appearance and transparency of coatings (Figure S2). 

### 3.3. Contact Angle and Surface Tension of WPU/Silica Coatings

**Figure 6:**
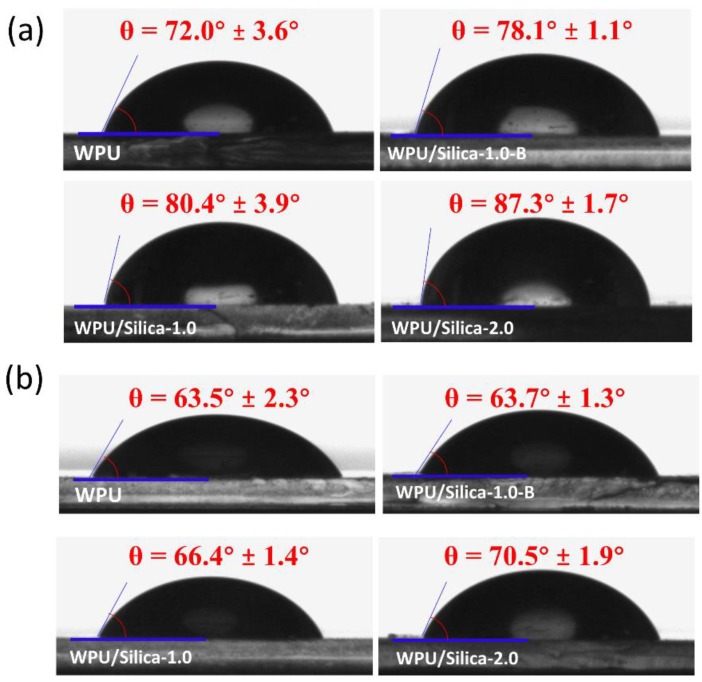
Contact angle of WPU and WPU/Silica coatings: (**a**) water and (**b**) glycol.

Figure 6 shows the water and glycol contact angles on coatings. When the content of alkyl-grafted silica increased from 0% to 2%, the contact angle (CA) of water on coating increased from 72.0° to 87.3° whilst the CA of ethylene glycol increased from 63.5° to 70.5°. This improved hydrophobicity of WPU/Silica coating may be because of the presence of nanosized silicon papillae on the surface creating the “lotus effect” [27]. Although WPU/Silica-1.0 has same silica content with WPU/Silica-1.0-B, its CA of water and ethylene glycol were larger than that of WPU/Silica-1.0-B. According to the Wenzel relation [28], surface roughness could lead to enhanced hydrophilicity of hydrophilic film. Since WPU/Silica-1.0 possesses smaller *R*_a_ than that of WPU/Silica-1.0-B, WPU/Silica-1.0 should show less hydrophilicity than WPU/Silica-1.0-B. Surface free energy (γ) is another important parameter to evaluate the properties of coating such as self-cleaning, adhesion, and anti-corrosion. Here, γ of coatings were calculated by substituting the contact angle values obtained using water and ethylene glycol in Owens-Wendt equation [29]. The results indicate the difference in the dispersive γ^d^ and the polar component γ^p^ of the WPU/Silica coatings (Table 1). These variations ultimately lead to the decreased γ after the addition of silica. As shown in Table 1, by increasing the content of silica from 0% to 2%, γ decreased from 32.87 to 21.54 mJ/m^2^, implying the enhanced hydrophobicity of WPU/Silica coatings.

### 3.4. Solvent Resistant Properties

**Figure 7:**
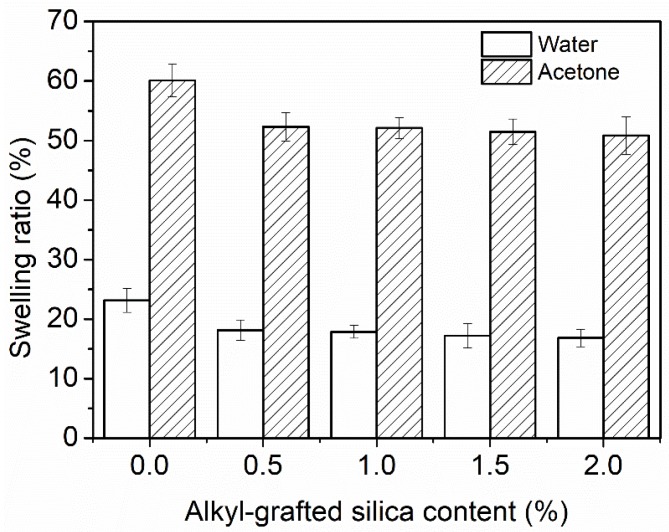
Water and acetone swelling ratio of coatings.

Figure 7 shows the water and acetone swelling of WPU coatings as a function of alkyl-grafted silica content. The incorporation of silica decreased the swelling ratio arises from both water and acetone. Specifically, the value of water and acetone swelling ratio decreased by 27% and 15%, respectively, as the content of silica was up to 2.0%. The results demonstrate that the presence of impermeable silica particles in WPU matrix reduces the swelling ratio and enhances the solvent resistance. This phenomenon may be attributed to that silica can work as cross-linking points in WPU matrix through interactions with polyurethane molecules [10,11]. Thus, the increased crosslinking density can make it difficult for solvent molecules to penetrate and diffuse into the coatings, therefore improving the solvent resistance. The enhancement of solvent resistance in other PU composite systems containing inorganic nanofillers such as nano-ZnO has also been reported [30].

### 3.5. Thermal Properties

**Figure 8:**
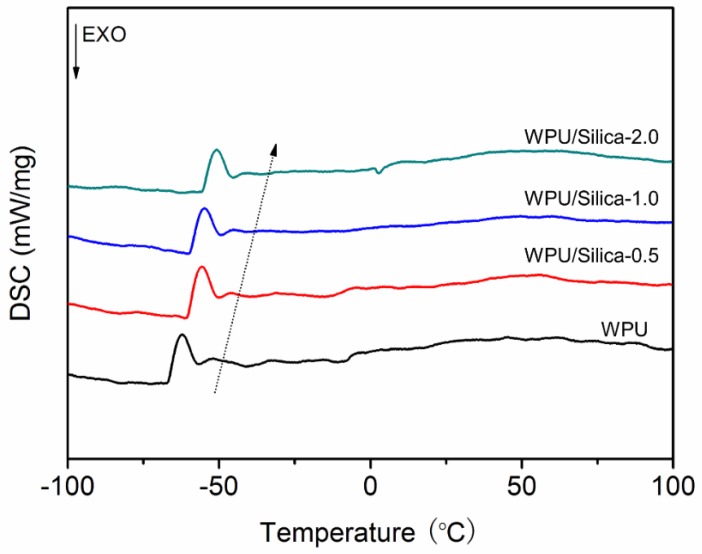
DSC curves of WPU and WPU/Silica coatings.

Previous research reported that the polar surface of fillers can influence the crystallization behavior of PU blocky segments [31]. Thus, DSC was used to investigate the interactions of alkyl-grafted silica with WPU. As presented in Figure 8, all investigated samples showed a pronounced glass transition behavior in the temperature range from −70 to −40 °C. This transition is due to the movement of soft-segment chains. Meanwhile, a broad endothermic peak can be observed at higher temperature, which is related to crystallization of hard segments. With increasing the alkyl-grafted silica content, the glass transition temperature (*T*_g_) of the soft-segment was increased whilst the endothermic peak of the hard-segment was expanded. Naik et al. [32] presented that weak interactions between filler and PU matrix would reduce the *T*_g_ of composite. Hassanajili et al. [33] suggested that organic-inorganic crystalline domains contribute to the increased *T*_g_ and melting point. Hence, in the case of WPU/Silica, the increases of endothermic peaks may be attributed to strong interfacial interaction between silica and polyurethane molecules, which on the one hand restrict the movement of soft segment and reduces the free volume of the chain movement [34], on the other hand create new hybrid domain with higher stiffness. Thus, the endothermic peak of hybrid domain may superimpose with that of the original polyurethane phase, causing the peak shifting to the high temperature region.

**Figure 9:**
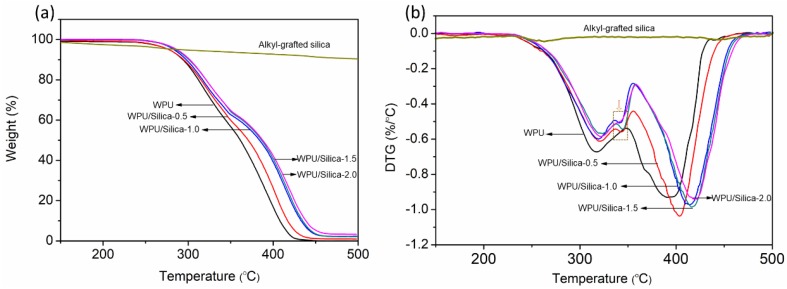
(**a**) TGA and (**b**) DTGA curves of alkyl-grafted silica, WPU, and WPU/Silica coatings.

Thermal stability is one of the references to determine the life of coatings applied in an environment with high temperature. Thermal degradation behavior for selected samples were demonstrated in TGA and DTGA as shown in Figure 9. The weight loss is summarized in Table 2. Apart from a slight weight loss at 200 °C, which can be assignable to the degradation of alkyl chains, no significant mass loss was observed when alkyl-grafted silica was heated up to 500 °C, indicating its intrinsic high thermal stability. As for WPU coatings, the first peak loss at 280 °C attributed to the degradation of hard-segment whilst the second major loss (about 60%) occurred from 360 °C corresponded to the decomposition of soft-segments as reported previously [15]. The incorporation of alkyl-grafted silica induced thermal stabilization of WPU/silica coatings. For example, with the load of 2.0% silica, the temperature for 50% weight loss was increased by about 30 °C compared to neat WPU. The improvement in thermal stability can be attributed to thermal insulation effect of silica lead to the delay of molecule chains degradation. Besides this, the interaction between the silica particles and molecules chains would lead to increasing potential energy needed for decomposition of polymer chains. It is also presented that the tortuous path effect of fillers in polymer matrix can retard the escape of volatile degradation products and char formation [35]. Moreover, compared to WPU, a new small peak in the temperature range of 335 to 355 °C appeared in the DTGA curves of WPU/Silica. With increasing the content of silica, the peak point shifted to higher temperature, which indicates the existing of a new hybrid domain.

### 3.6. Mechanical Properties

**Figure 10:**
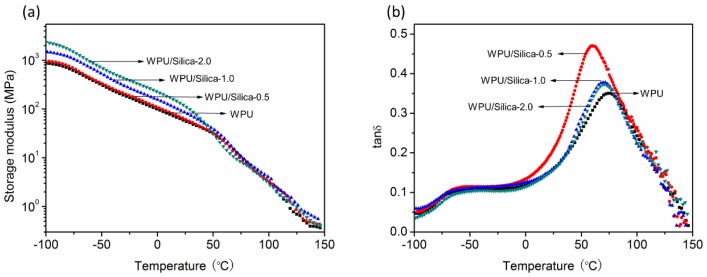
(**a**) Storage modulus (*E*′) and (**b**) loss factor (tanδ) of WPU and WPU/Silica coatings.

Figure 10a demonstrates temperature dependence of storage modulus (*E*′) for different samples. It can be seen that the introduction of alkyl-grafted silica significantly increased the *E*′ of WPU/Silica coatings in the region where hard-segments were in the glassy state (Figure 10a). This enhanced stiffness indicates that the presence of silica preferentially resided in the soft segment, which hinders the macromolecular chain segmental movements and results in tougher hybrid coatings. Figure 10b shows that WPU/Silica coatings had broad peaks of loss factor (tanδ) at low temperature (−45~−60 °C) and high temperature regions (0~125 °C), which corresponded to the glass transition temperature of soft-segment (*T*_g-s_) and hard-segment (*T*_g-h_), respectively. This temperature range of transition is consistent with the results of DSC. Compared to neat WPU, the *T*_g-h_ of hard-segment for WPU/Silica first decreased at 0.5% silica loading and then gradually shifted to higher temperature region when further increasing silica content. This kind of silica-content determined variations of *T*_g_ are also reported in other literature [36]. On one hand, the incorporation silica particle may weaken the interactions between the hard-segment and increase the free volume of molecular chains; on the other hand, silica can be compounded with polyurethane segments to form hybrid domains with high modulus. These two effects are in a competitive relationship. Hence, the results indicate that when the alkyl-grafted silica content is at a low level (0.5%), the influence of the former dominates, leading to the decreased of *T*_g-h_. After increasing the silica content (>1%), the effect of the latter governs, thus increasing the *T*_g-h_. In the case of soft-segment, *T*_g-s_ steadily shifted to the higher temperature region with silica content as evidenced in DSC results. This may be because soft-segments have long molecular chains with inherent mobility which would be easily restricted in the vicinity of silica particles due to the entanglement between alky chain on surface of silica and molecules chains. The peak values and width of loss factor (tanδ) can be used to predict the damping behavior which is important for special coatings with vibration damping, thermal insulation, and sealing performance. As shown in Figure 10b, by incorporating silica, peak values of tanδ of coatings increased, indicating the enhanced damping capacity. This verifies that damping properties of WPU can be enhanced by controlling the content of incorporated silica.

**Figure 11:**
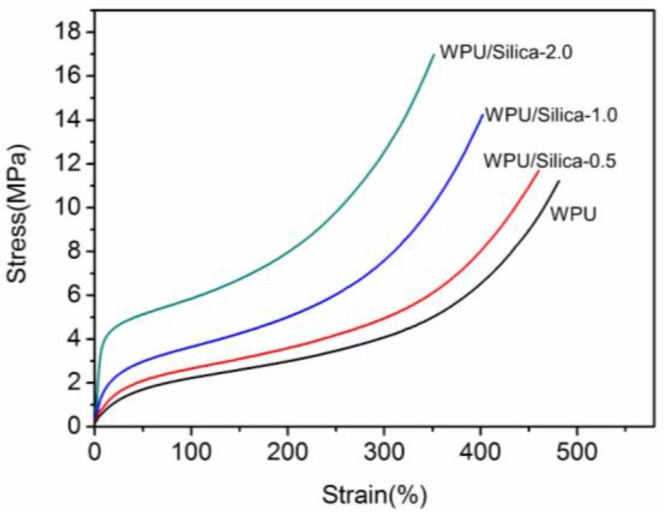
Stress-strain curves of WPU and WPU/Silica coatings.

Representative stress-strain curves for WPU/Silica coatings are shown in Figure 11. When the content of alkyl-grafted silica increased from 0% to 2.0%, the breaking strength of WPU/Silica coatings increased from 11.10 to 17.2 MPa. The results indicate that the presence of silica particle can act as a physical netpoint, contributing to the high strength of WPU/Silica coatings. Nevertheless, the presence of silica led to some decline of elongation property. By increasing silica content from 0% to 2%, the strain at the break decreased from 480% to 350%, implying the reduced ductility of coatings which may result from the restriction effect of silica on the mobility of molecular chains [9].

## 4. Conclusions

After addition of alkyl-grafted silica, the average particle size of waterborne polyurethane (WPU) dispersions enlarged whilst the particle size distribution broadened. The emulsion viscosity increased with increasing content of alkyl-grafted silica. Results verify that the in situ incorporation of alkyl-grafted silica does not impair the synthesis of high solid content of WPU. FTIR study confirmed the successful incorporation of alkyl-grafted silica into polyurethane matrix and their preferred molecular interactions. Evidenced by AFM and SEM results, alkyl-grafted silica showed good dispersion and compatibility with WPU. The nanosized silica papillae on surface contributed to the enhanced surface roughness and hydrophobicity of coatings. Solvent resistance of WPU coatings was improved with existence of alkyl-grafted silica particles. *T*_g_ of WPU/Silica coatings shifted to the higher temperature region with increasing silica content. The combination of thermal insulation ability and tortuous path effect of silica particles led to better thermal stability. Compared to neat WPU, WPU/Silica coatings showed higher storage modulus, Young’s modulus, and tensile strength without much loss of ductility. Hence, the resultant hybrid coatings present superior comprehensive properties, which will contribute to the coating applications of WPU with high solid content.

## Figures and Tables

**Table 1:** Surface free energy of WPU and WPU/Silica coatings.

Sample	Surface Free Energy (mJ/m^2^)		
	γ^d^	γ^p^	γ
WPU	5.23 ± 0.88	27.64 ± 4.43	32.87 ± 3.55
WPU/Silica-1.0-B	9.84 ± 0.51	16.80 ± 0.76	26.64 ± 0.78
WPU/Silica-1.0	9.57 ± 2.16	15.70 ± 4.68	25.27 ± 2.52
WPU/Silica-2.0	12.17 ± 0.93	9.37 ± 1.01	21.54 ± 1.02

**Table 2:** TG data of WPU and WPU/Silica coatings.

Samples	Temperature (°C)			
	30% Weight Loss	50% Weight Loss	Tmax_1_	Tmax_2_
WPU	326.3	361.8	315.8	393.2
WPU/Silica-0.5	331.0	370.1	319.8	404.0
WPU/Silica-1.0	334.8	383.4	320.0	413.2
WPU/Silica-1.5	339.5	386.3	321.8	416.5
WPU/Silica-2.0	339.5	388.2	322.6	419.8

Abbreviations: Tmax_1_, temperature at first major weight loss; Tmax_2_, temperature at first major weight loss.

## Supplementary Materials

The following are available online at http://www.mdpi.com/2073-4360/10/5/514/s1, Figure S1: Phase images of WPU and WPU/Silica coatings, Figure S2: Photos of WPU and WPU/Silica coatings.
