# Supplementary material for: In-Situ Incorporation of Alkyl-Grafted Silica into Waterborne Polyurethane with High Solid Content for Enhanced Physical Properties of Coatings
Source: Polymers (Basel). 2018 May 9;10(5):514. doi: 10.3390/polym10050514 (PMC6415436; doi:10.3390/polym10050514)
Supplement: Supplementary file 1 [file polymers-10-00514-s001.pdf]

In situ incorporation of alkyl-grafted silica into waterborne polyurethane with high solid content for enhanced physical properties of coatings

Yanting Han[1], Jinlian Hu[1]*, Zhongyin Xin[2]

1. Institute of Textiles & Clothing, The Hong Kong Polytechnic University, Hung Hom, Kowloon 999077, Hong Kong, China

2. National Engineering Laboratory for Clean Technology of Leather Manufacture, Sichuan University, Chengdu, Sichuan, China

* Correspondence: jinlian-hu@polyu.edu.hk

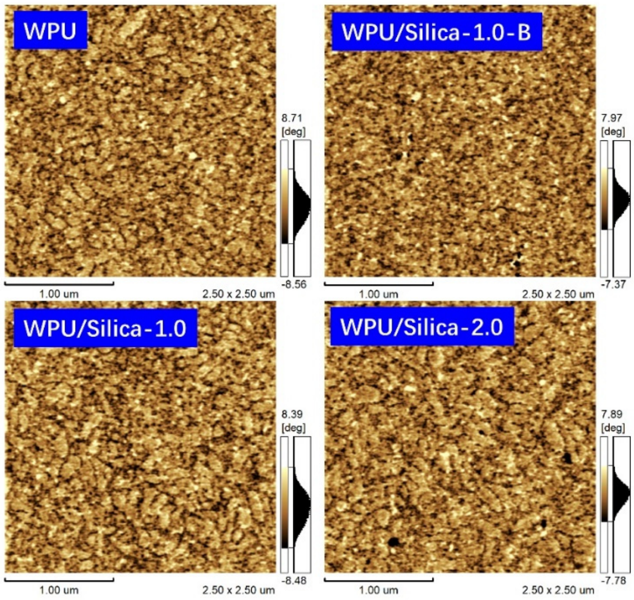

Figure s1. Phase images of WPU and WPU/Silica coatings.

Microphase structure is essential for mechanical properties of PU coatings. It is known that hard-segment and soft-segment phases contribute to the strength and flexibility of polyurethane, respectively. The AFM phase images of WPU (Figure 1) showed a typical micro-phase separation structure in which the bright domains corresponded to the microphase of the hard-segments while the dark domains attributed to the soft-segments. With incorporation of alkyl-grafted silica, the bright areas increased because the silica particles were embedded in the polymer molecular chains, forming hybrid domains with higher modulus.

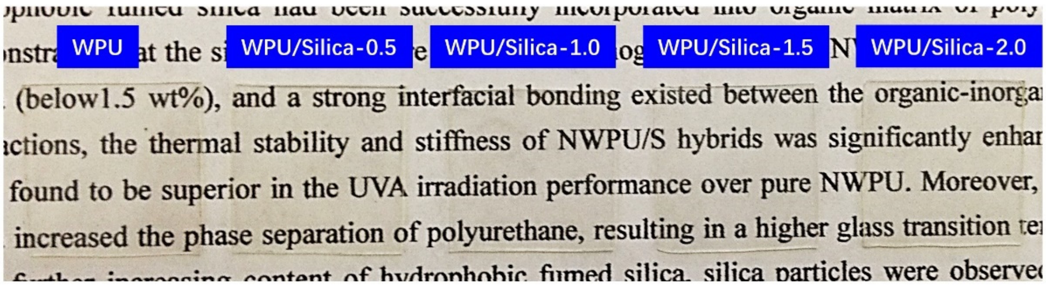

Figure s2. Photos of WPU and WPU/Silica coatings

The photos of WPU and WPU/Silica coatings were taken by putting them on a piece of printed paper. As shown in Figure 2, there was no apparent specular reflection on all samples. Besides this, the incorporation of alkyl-grafted silica had little influence on transparency of coatings when the content of silica is less than 2%. This is consistent with research conducted by Eslami et al [1], where they found the addition of nano-silica below 2% would not result in a decrease in the degree of the transparency.

Reference:

1. Eslami, R.; Bagheri, R.; Hashemzadeh, Y.; Salehi, M. Optical and mechanical properties of transparent acrylic based polyurethane nano silica composite coatings. *Progress in Organic Coatings* 2014, *77*, 1184-1190.
